# Editorial for the Special Issue “Editorial Board Members’ Collection Series: Aerosol Particles”

**DOI:** 10.3390/toxics13070568

**Published:** 2025-07-05

**Authors:** Matthias Karl, Yuan Cheng

**Affiliations:** 1 Department of Coastal Environmental Chemistry, Helmholtz Zentrum Hereon, 21502 Geesthacht, Germany; 2 State Key Laboratory of Urban Water Resource and Environment, School of Environment, Harbin Institute of Technology, Harbin 150090, China; ycheng@hit.edu.cn

## 1. Introduction

Aerosol particles play a prominent role in several of our modern-day societal challenges, such as the development of sustainable cities, clean energy production, and combating climate change. Aerosol particles are a mixture of solid, liquid/solid, and liquid particles suspended in the air that vary in size, composition, and origin. Anthropogenic activities contribute significantly to emissions of particles through combustion processes in transportation, industrial activities, power plants, and residential heating. In addition, secondary aerosols form through chemical reactions in the atmosphere, such as the transformation of gaseous pollutants into particulate matter. The mass concentration of atmospheric aerosol particles (particulate matter; PM) has been systematically used by epidemiologists as an indicator of exposure to air pollution, connecting airborne PM with a wide variety of health impacts. Exposure to fine particles (PM_2.5_, with an aerodynamic diameter of ≤ 2.5 μm) can cause premature death, as well as harmful effects on the cardiovascular system, and has been linked to respiratory diseases. Despite the proven health risks related to PM_2.5_, it remains difficult to explain the health effects associated with aerosol particles through just one parameter, mainly because particles are formed by a complex mixture of chemicals and their sizes span over four orders of magnitude, from a few nanometers to tens of micrometers. In recent decades, many efforts have been undertaken to advance our understanding of the sources, occurrence, chemical composition, and toxicity of aerosol particles. New technologies for reducing particle emissions from mobile sources, mainly targeting diesel engines, have been developed and implemented in countries worldwide.

## 2. An Overview of Published Articles

The five articles contributed to this Special Issue address a wide variety of aspects related to aerosol particles, encompassing emission sources, atmospheric transformation and dispersion, and the assessment of associated environmental and health risks, as well as methodological aspects of measurements, emission control, and health protection.

The health effects associated with black carbon (BC) are consistent with those associated with PM_2.5_. Black carbon, a product of the incomplete combustion of fossil fuel, biomass, and biofuels, has an impact on both air quality and climate. BC is the principal light-absorbing component of atmospheric aerosol particles that heat the atmosphere. The morphology, size, and composition of freshly emitted BC changes during transport in the atmosphere due to transformation processes, whereby the primary part becomes increasingly embedded in other organic and inorganic components. This process, known as BC aging, affects the light absorption and refractory properties of aerosol particles. The accurate quantification of BC still poses a significant challenge, impeding the comprehensive assessment of its impacts on human health and climate. Contribution 1 reviewed three quantitative methods for measuring BC, Thermal Optical Analysis (TOA), the Optical Method, and Laser-Induced Incandescence (LII), which utilize distinct physical properties (thermal and optical) to quantify black carbon. Generally, BC measured using the TOA is referred to as elemental carbon (EC) and BC measured through optical methods is referred to as equivalent black carbon (eBC), while BC determined using LII is referred to as refractory black carbon (rBC). The review elucidates the underlying relationships and fundamental disparities among the different measured BC quantities and concludes with recommendations for measurement protocols and future research.

Potentially toxic compounds, including bioavailable transition metals and polycyclic aromatic hydrocarbons (PAHs), attached to the particle surface, can increase the toxicity risks of aerosol particles and may even determine the adverse effects of combustion particles on human health. PAHs are ubiquitous persistent organic pollutants characterized by biological enrichment, hydrophobicity, and resistance to biodegradation. While the lighter PAHs, comprising two or three benzene rings, mainly partition to the gas phase, the heavier congeners are predominantly found in the particulate phase and generally have the highest carcinogenic and mutagenic potencies. PAHs resulting from traffic emissions and the incomplete combustion of fossil fuel or wood are subsequently removed from the atmosphere through dry and wet deposition and become associated with road dust or incorporated into soil. The work reported in Contribution 2 analyzed the concentration of various PAHs in road dust, green belt soil, and parking lot dust samples to obtain a comprehensive understanding of the occurrence and origin of these compounds. The abundance of carcinogenic PAHs revealed that the toxic potency of the samples in the green belt soil was higher than in the road dust and the parking lot dust. The diagnostic ratios of PAHs showed that petroleum, petroleum combustion, and biomass/coal combustion were the major sources of PAHs in road environments.

Long-term oil extraction activities lead to the accumulation of high PAH concentrations in the nearby environmental media. The authors of Contribution 3 investigated the spatial distribution, sources, and air–soil exchange of PAHs and alkylated PAHs in an oilfield area. Source apportionment revealed that coal/biomass combustion was the main contributor to atmospheric polycyclic aromatic compounds in urban, suburban, and agricultural areas, while crude oil production primarily affected the industrial and oilfield area. The fugacity ratios suggest that the soil is generally a source of lighter PAHs but acts as a sink for heavier PAHs. The lifetime cancer risk posed by PAHs in the soil of the oilfield area was about an order of magnitude higher than that in the surrounding region.

The smallest size fraction of PM, ultrafine particles (PM_0.1_, UFPs) with an aerodynamic diameter of ≤ 0.1 μm, can penetrate deep into the lungs, crossing from the alveoli into the blood stream where they can circulate in the human body. The inhalation of UFPs is associated with inflammation and oxidative stress, which are starting points for further diseases. Oxidative stress occurs when concentrations of reactive oxygen species (ROS) within human lung cells overwhelm cellular antioxidant defenses. The capacity of particles to produce ROS with the simultaneous depletion of antioxidants is defined as the oxidative potential. Epidemiologic studies using acellular assays have found that exposure to PM with high oxidative potential affects cardiorespiratory health.

Maritime transport emerges as an important source of UFP pollution, with consequences for the health of people living in port cities. Most particles emitted in fresh ship exhaust are in the ultrafine size range; thus, number-based concentrations of aerosol particles represent a better metric for the impacts of shipping emissions than the PM mass. The authors of Contribution 4 applied a coupled chemistry transport modeling system on regional and local scales to quantify the impact of shipping on UFP and secondary organic aerosol concentrations in a port city. The estimated oxidative potential of daily mean particulate organic matter related to shipping activities was lower than previously reported. The lower oxidative potential is explained by the low share of ships using heavy fuel oil during stopover in the port.

The severe acute respiratory syndrome coronavirus 2 (SARS-CoV-2) pandemic has highlighted the importance of transmission though ambient aerosol particles, as the virus can remain viable on aerosols for hours. The usefulness of wearing face masks has been a topic of public discussions and led to the emergence of evaluation studies on the efficiency of masks in capturing aerosols. The authors of Contribution 5 tested a range of filter pieces from commonly used masks through an experimental matrix with humidity control to determine the relationship between filtration efficiency and mask surface potential. This study highlights the critical role of electrostatic interactions in capturing nanometer-sized particles, which is reflected in the surface potential of masks. Relative humidity conditions strongly impact the filtration efficiency and charge distributions of the masks.

## 3. Future Development Prospects

For the effective management and reduction of particle pollution, more measurement and modeling studies are needed to characterize emissions, especially from unregulated combustion sources, and the processes that control the transformation of aerosol particles in the atmosphere. The scope of black carbon measurements should be broadened under diverse atmospheric conditions through the deployment of multiple co-located instruments. Modeling techniques such as computational fluid dynamics, chemistry transport models, and machine learning systems are useful methods in quantifying the spatial distribution of particulate pollutants and assessing the potential for adverse health outcomes. Comprehending the filtration efficiency and mechanism of face masks in relation to airborne ultrafine particles and the development of low-cost sensors for the densification of monitoring networks for ultrafine and fine particulate matter are growing areas of interest for health protection in public spaces and occupational settings.

